# Supplementary material for: Integrated Transcriptomic and Metabolomic Analysis Revealed Abscisic Acid-Induced Regulation of Monoterpene Biosynthesis in Grape Berries
Source: Plants (Basel). 2024 Jul 5;13(13):1862. doi: 10.3390/plants13131862 (PMC11243831; doi:10.3390/plants13131862)
Supplement: Supplementary file 1 [file plants-13-01862-s001.zip › plants-3080168-supplementary.pdf]

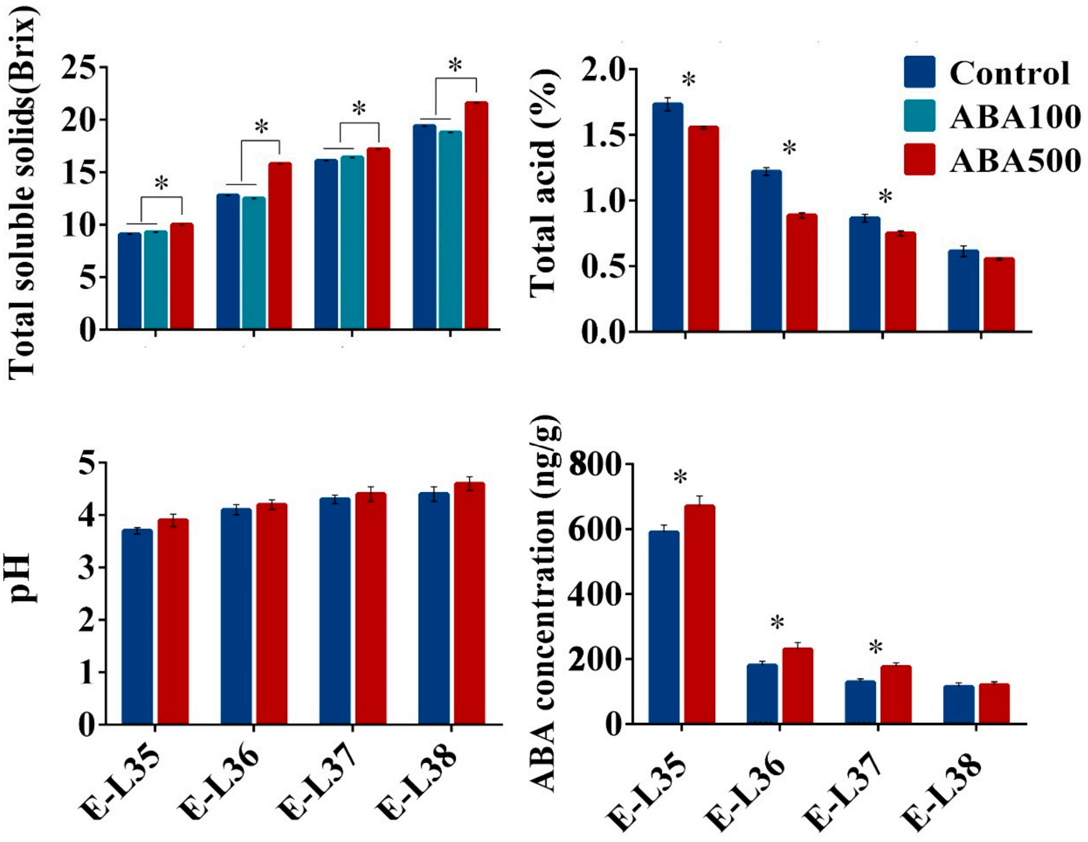

Figure S1. Physicochemical indexes of grape berries from ABA-treated control grapes at the four phenological stages. Data are presented as mean ± standard error (n=3). * indicate significant differences (Duncan' s multiple range test, p≤ 0.05)

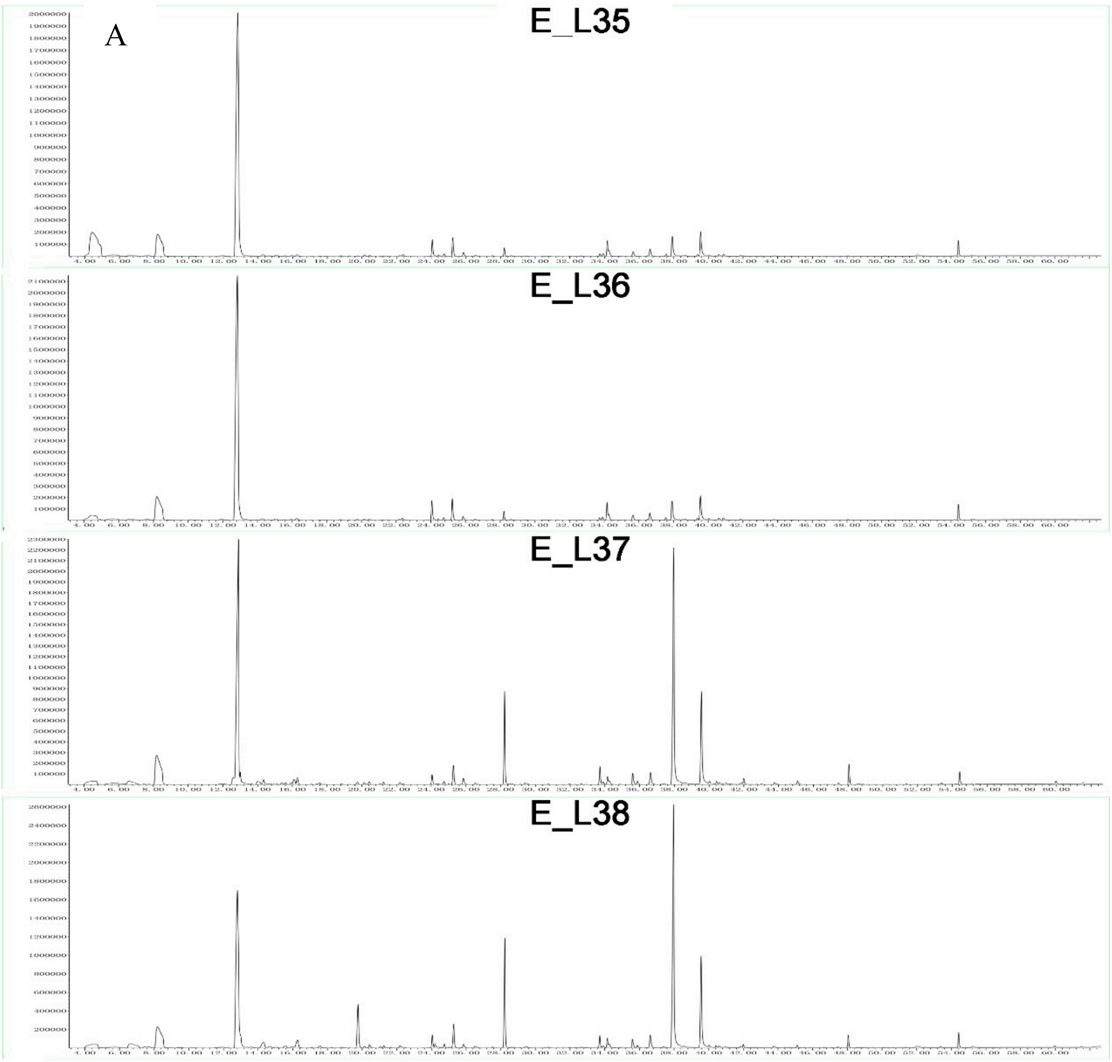

A
E_L35
E_L36
E_L37
E_L38

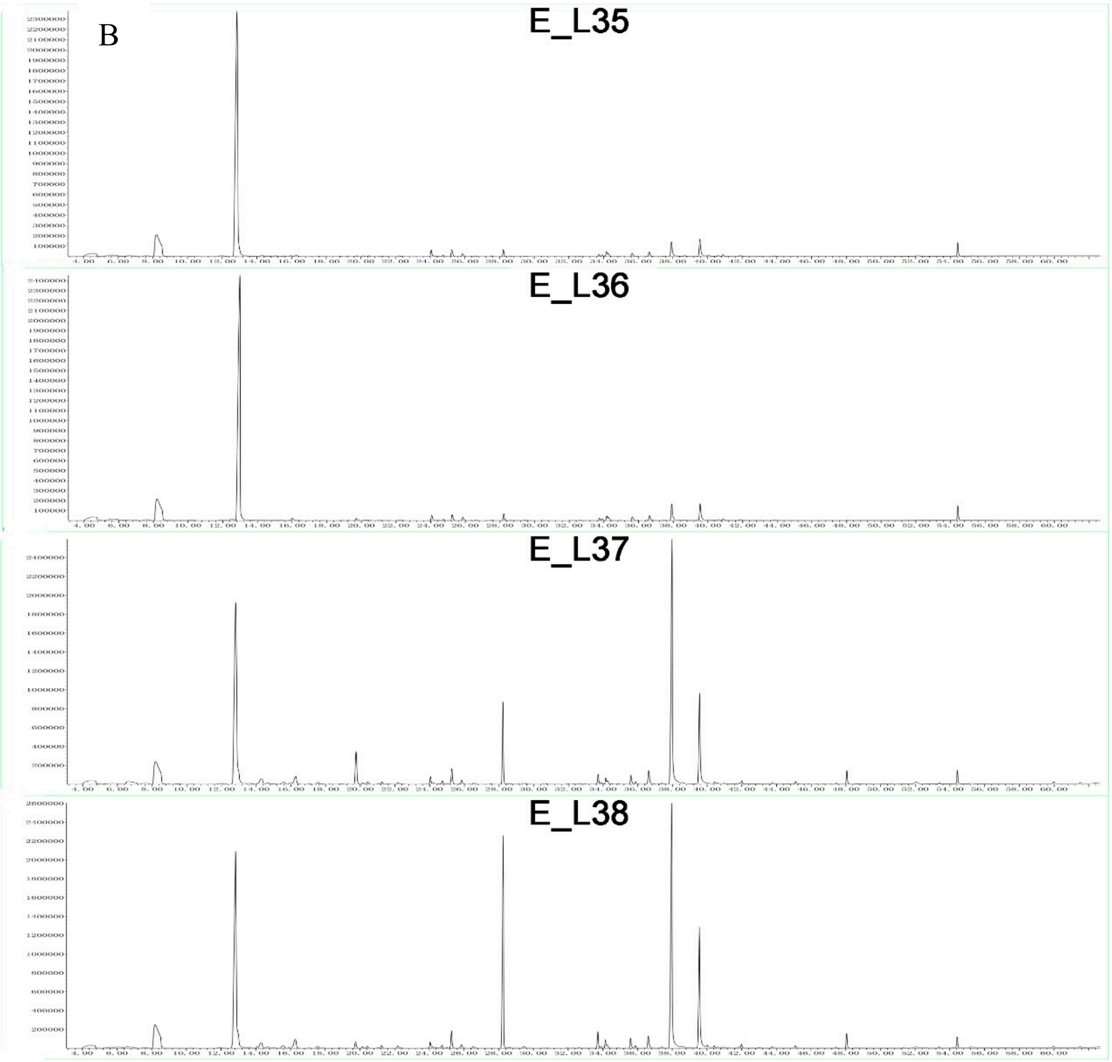
B
E_L35
E_L36
E_L37
E_L38

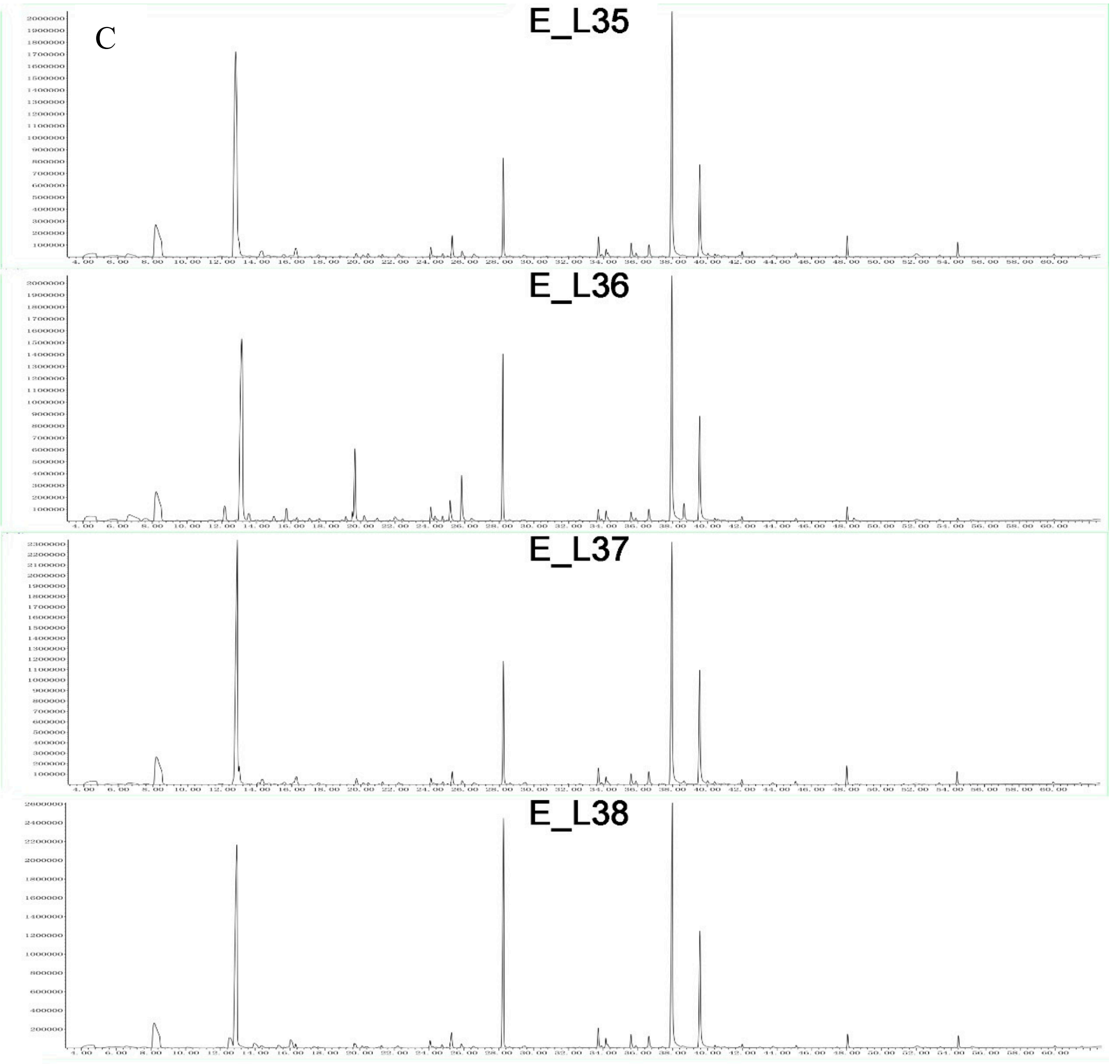
C
E_L35
E_L36
E_L37
E_L38

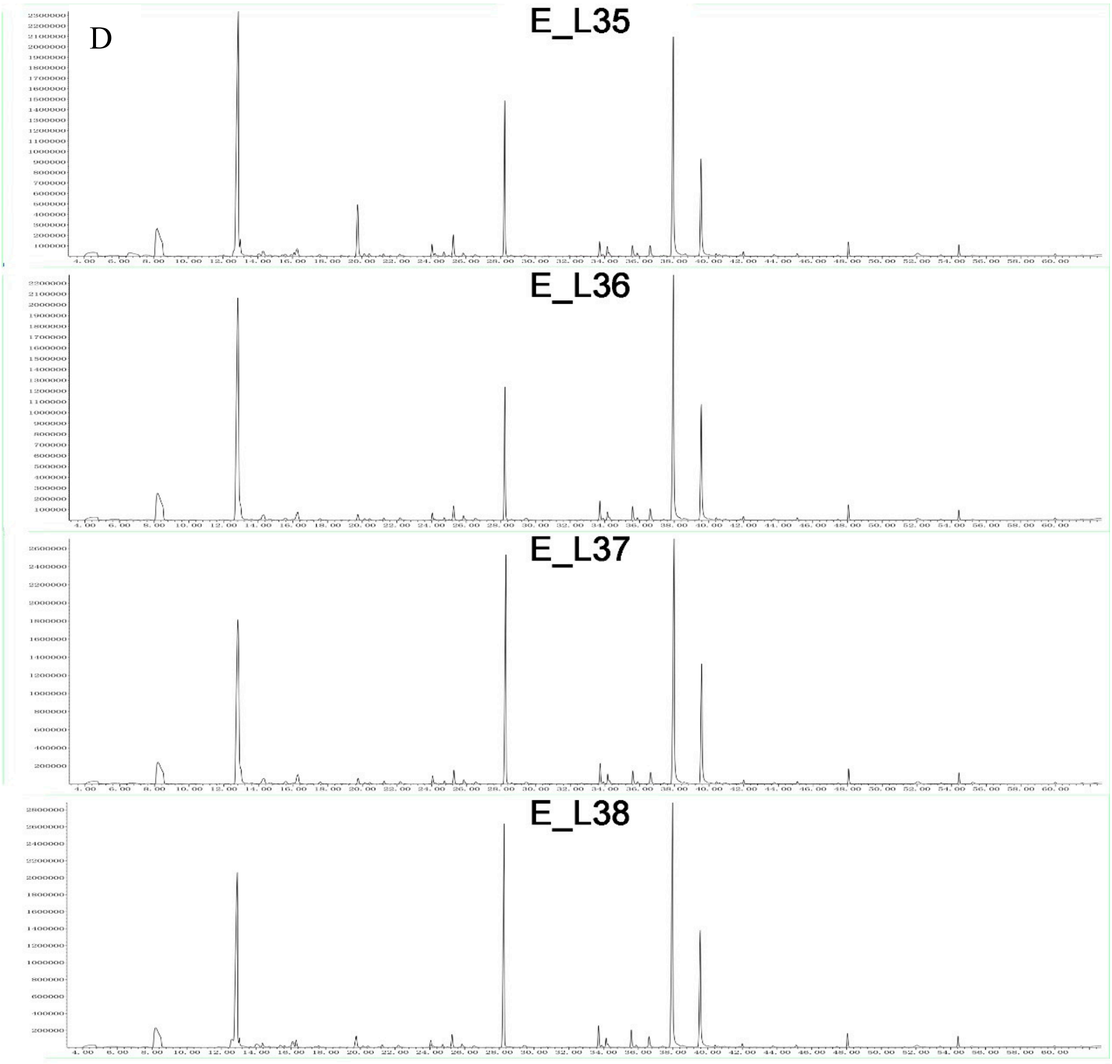

Figure S2. Chromatograms of HS- SPME/GC–MS analysis for monoterpenes.

Free monoterpenes in grape berries at four developments as control (A); Bound monoterpenes in grape berries at four developments as control (B); Free monoterpenes in grape berries at four developments under ABA treatment (C); Bound monoterpenes in grape berries at four developments under ABA treatment (D).

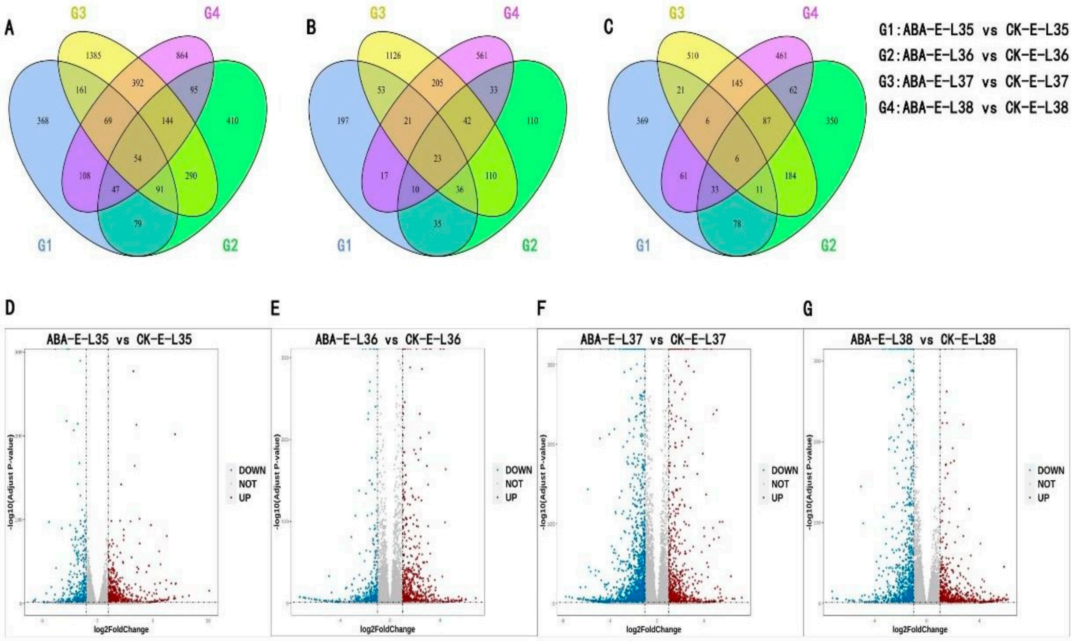

Figure S3. Summary of the differential expressed genes (DEGs) found using RNA-seq sequencing of grapes under the administration of abscisic acid (ABA) compared to the control (CK) groups. The quantities of overall differentially expressed genes (DEGs) (A), upregulated DEGs (B), and downregulated DEGs (C) are visually represented using Venn diagrams. A volcano plot depicting the differential expression of genes between ABA and CK treatments at four stages (D, E, F, and G). The abscissa in the differential expression volcano map signifies the logarithm of the multiple differential expression of a gene in the two samples. Each pixel on the map corresponds to a distinct gene. The y-axis displays the negative logarithm of statistically significant changes in gene expression. The blue dots correspond to downregulated differentially expressed genes (DEGs), the red dots correspond to upregulated DEGs, and the gray dots correspond to genes that are not differently expressed.

Table S1 GenBank accession number and primers for quantitative real-time PCR (qPCR).

Gene ID	Gene name	Forward (5’ to 3’)	Reverse (5’ to 3’)
VIT_05s0020g02130	*VvDXS*	GGTGGTGCATGATGTAGATTTG	CAAGCCATGAAAGTGACATCAA
VIT_18s0001g06060	*VvGT14*	GCATATTTTCTGACGCTGTCAT	TGTAAAACCCTTGTCGATGAGA
VIT_05s0051g00670	*VvNCED*	CAAACTATCGGAAATGGTTCGG	ATCCACTGAATTCTGGATTCGT
VIT_04s0044g00270	*VvZEP*	AAATGTAAGCAAAGGGACGATG	CACCCTGATATATATGACCCCG
VIT_00s0271g00030	*VvTPS*	AGAGACAGTATATGAAAGCCGG	CTCAATAGCCGAAAACGAAGAG
VIT_08s0007g05360	*VvABA2*	TCTACATTGCGGACGCTTATAT	CTGAAATATACCGCTCCGTTTG
EC969944	*VvActin*	CTTGCATCCCTCAGCACCTT	TCCTGTGGACAATGGATGGA
CB975242	*VvGAPDH*	TTCTCGTTGAGGGCTATTCCA	CCACAGACTTCATCGGTGACA

Table S2. Sequencing Data Statistics.

Sample name	Total reads	Total mapped	Multiple mapped	Unique mapped	Non-splice reads	Splice reads
ABA-E-L35-1	77610916	71786508(92.50%)	2210432(2.85%)	69576076(89.65%)	41727490(53.76%)	27848586(35.88%)
ABA-E-L35-2	75966380	70827811(93.24%)	2192140(2.89%)	68635671(90.35%)	40809401(53.72%)	27826270(36.63%)
ABA-E-L35-3	102695590	95115932(92.62%)	2907707(2.83%)	92208225(89.79%)	61151181(59.55%)	31057044(30.24%)
ABA-E-L36-1	66257770	56558228(85.36%)	1628098(2.46%)	54930130(82.90%)	33752700(50.94%)	21177430(31.96%)
ABA-E-L36-2	72010808	61657591(85.62%)	1775379(2.47%)	59882212(83.16%)	36885963(51.22%)	22996249(31.93%)
ABA-E-L36-3	74045888	62919897(84.97%)	1877250(2.54%)	61042647(82.44%)	38049294(51.39%)	22993353(31.05%)
ABA-E-L37-1	80055312	74364056(92.89%)	2408808(3.01%)	71955248(89.88%)	43890901(54.83%)	28064347(35.06%)
ABA-E-L37-2	77849440	72059595(92.56%)	2372114(3.05%)	69687481(89.52%)	42932691(55.15%)	26754790(34.37%)
ABA-E-L37-3	65093884	60591910(93.08%)	1922249(2.95%)	58669661(90.13%)	35769648(54.95%)	22900013(35.18%)
ABA-E-L38-1	79904370	74994553(93.86%)	2365293(2.96%)	72629260(90.90%)	44287209(55.43%)	28342051(35.47%)
ABA-E-L38-2	77924092	73124157(93.84%)	2328316(2.99%)	70795841(90.85%)	42997280(55.18%)	27798561(35.67%)
ABA-E-L38-3	78522640	73393439(93.47%)	2282255(2.91%)	71111184(90.56%)	43160383(54.97%)	27950801(35.60%)
CK-E-L35-1	84775718	77932600(91.93%)	2394121(2.82%)	75538479(89.10%)	45755844(53.97%)	29782635(35.13%)

CK-E-L35-2	95327984	88697096(93.04%)	2790981(2.93%)	85906115(90.12%)	51452628(53.97%)	34453487(36.14%)
CK-E-L35-3	85139260	79363764(93.22%)	2473912(2.91%)	76889852(90.31%)	46239909(54.31%)	30649943(36.00%)
CK-E-L36-1	78331142	73322361(93.61%)	2229490(2.85%)	71092871(90.76%)	42549726(54.32%)	28543145(36.44%)
CK-E-L36-2	81588516	76504834(93.77%)	2297957(2.82%)	74206877(90.95%)	44274604(54.27%)	29932273(36.69%)
CK-E-L36-3	89571980	83840350(93.60%)	2563886(2.86%)	81276464(90.74%)	49044189(54.75%)	32232275(35.98%)
CK-E-L37-1	83033188	71730589(86.39%)	2380994(2.87%)	69349595(83.52%)	42129115(50.74%)	27220480(32.78%)
CK-E-L37-2	74828824	64503314(86.20%)	2069861(2.77%)	62433453(83.44%)	38296905(51.18%)	24136548(32.26%)
CK-E-L37-3	65425076	56449416(86.28%)	1876911(2.87%)	54572505(83.41%)	33254981(50.83%)	21317524(32.58%)
CK-E-L38-1	64557962	53378204(82.68%)	1679769(2.60%)	51698435(80.08%)	31277717(48.45%)	20420718(31.63%)
CK-E-L38-2	66832480	54956468(82.23%)	1731957(2.59%)	53224511(79.64%)	32264913(48.28%)	20959598(31.36%)
CK-E-L38-3	68205746	56368672(82.65%)	1744848(2.56%)	54623824(80.09%)	32497358(47.65%)	22126466(32.44%)

Table S3. Number of genes in each WGCNA module

Module	Gene Number
darkred	1235
brown	781
black	712
darkgreen	390
greenyellow	186
tan	160
red	146
magenta	142
purple	140
midnightblue	93
grey60	85
lightgreen	83
darkturquoise	69
darkgrey	66

darkorange	49
skyblue	43
steelblue	42
